# Development of an HPLC method for determination of pentachloronitrobenzene, hexachlorobenzene and their possible metabolites

**DOI:** 10.1186/1472-6769-11-2

**Published:** 2011-11-23

**Authors:** Fazlurrahman Khan, Dhan Prakash, RK Jain

**Affiliations:** 1 Institute of Microbial Technology (CSIR), Sector 39-A, Chandigarh-160036, India

## Abstract

**Background:**

Pentachloronitrobenzene (PCNB) and hexachlorobenzene (HCB) are highly toxic and widespread in every environmental compartment. Some of metabolic products such as amino/nitro containing chlorinated aromatic compounds can be determined by gas chromatography coupled with electron capture detector (GC-ECD). However, it is difficult to identify some of chlorophenolic and chloroquinolic intermediates produced from PCNB and HCB by the above mentioned technique. Therefore, for analysis of these compounds and their metabolites, we have developed a high performance liquid chromatography (HPLC) based method.

**Results:**

The extraction of PCNB and HCB from soil and minimal salt medium was carried out with ethyl acetate and hexane respectively with good recoveries (98% for PCNB and 97% for HCB). The validation of the proposed extraction and HPLC method was done by analysis of PCNB and HCB biodegradation and their metabolites identification from anaerobic enriched soil samples.

**Conclusion:**

A rapid, sensitive and simple HPLC based analytical method was developed for the analysis of PCNB, HCB and their possible intermediates.

## Background

Farmers use pesticide (herbicides, insecticides, fungicides, molluscicides, rodenticides, acaricides and nematicides) for the protection of crop [1]. Chlorinated aromatic compounds (CACs) have been widely used in industrial, agricultural and domestic products such as pesticides, soil fumigants, disinfectants, toilet deodorants, solvents, and also used as precursors for the production of dyes [2,3].

As a result of worldwide extensive use, CACs are widespread and released as environmental pollutants in coastal marine sediments, freshwater lake sediments, sewage sludge, waste water, groundwater, rivers and estuaries, and soils [4-6]. Due to their persistence, toxicity, and bioaccumulation potential, CACs are subject to regulation in most of the developed countries [7]. The main environmental impact of pesticide is caused by their mobilization and transport from agriculture fields to pollute soils, sediment, water and also percolating through the soil and reaching the ground water [8-11].

Pentachloronitrobenzene (PCNB) is an important group of organochlorine fungicide which has been widely used either for seed dressing or for soil treatment to control a wide range of phytopathogenic fungi in crops [12,13]. Hexachlorobenzene (HCB) is also used as a fungicide and is a side product of industrial emission related to the manufacture of chlorinated solvents and pesticides [14-16]. Both organochlorine fungicide (PCNB and HCB) are widespread and found in every environmental compartment i.e. soil, water, and air [17,18]. Lipophilic nature of these compounds results in their bioaccumulation at different trophic levels via the food chain, thereby increasing risk to different living beings [19,20]. The accumulation of organochlorine pesticide is due to their resistance to both biotic and abiotic degradation in environmental condition [21]. Public concern has prompted the need for removal of these contaminants from the environment. Therefore, there has been an increase in effort to remove PCNB, HCB and their possible intermediates from the environment either by abiological or biological means during the past decades. The biotransformation products of PCNB include pentachloroaniline (PeCA), tetrachloroaniline (TeCA), trichloroaniline (TCA), dichloroaniline (DCA), chloroaniline (CA), pentachlorophenol (PCP), pentachlorothiophenol, pentachlorothioanisole and pentachloromethyl-phenyl sulfoxide [22-25]. The biotransformation products of HCB are pentachlorobenzene (PeCB), tetrachlorobenzene (TeCB), trichlorobenzene (TCB), dichlorobenzene (DCB), pentachlorophenol (PCP), tetrachlorohydroquinone (TeCH) and dichlorohydroquinone (DiCH) [14-16]. Some of these polychlorinated and their nitro or amino derivatives metabolites such as PeCB, TeCB, TCB, DCB, PeCA, TeCA, TCA, DCA and CA can be determined by gas chromatography coupled with electron capture detector (GC-ECD) [26-29]. However, it is difficult to identify some of chlorophenolics and chloroquinolics intermediates such as PCP, tetrachlorophenol (TeCP), trichlorophenol (TCP), dichlorophenol (DCP) and TeCH, DiCH by GC due to their relative polarity, low vapour pressure, chemical reactivity, causing adsorption and tailing of the chromatographic peaks [30,31]. Therefore, for determination of these CACs and chemically different nature (polar/ionic) of the putative intermediates, there is a need to develop simple, quick extraction and analytical method. In the present study we report a high performance liquid chromatography (HPLC) method for determination of PCNB, HCB and their possible metabolites.

## Results

### Solvents selection for extraction

The solvents were selected for extraction of PCNB, HCB and their possible metabolites by dissolving increasing concentrations of compounds up to their maximum solubility into different organic solvents. Solubility tests revealed that all compounds were best soluble in ethyl acetate whereas HCB was only soluble in hexane. The selected solvents were used for the determination of extraction efficiency of PCNB, HCB and their possible metabolites.

### Extraction efficiency and quantification

Quantitative determination of PCNB and HCB were performed by comparing peak areas of spiked soil with that of standards. Calibration graphs were constructed by plotting peak areas versus PCNB and HCB concentrations. Five working concentrations of PCNB and HCB compounds equivalent to 0.5, 1.0, 2.0, 3.0 and 4.0 μg were added to 1 ml ethyl acetate and hexane respectively and analyzed in triplicate. A best linearity was found from the concentrations 0.5-4.0 μg ml^-1 ^as indicated by correlation coefficient of 0.998 for both PCNB and HCB. Recoveries were studied by using five concentrations from 0.5, 1.0, 2.0, 3.0 to 4.0 μg ml^-1^. The mean recoveries and relative standard deviation (R.S.D.) of PCNB and HCB are presented in the Table 1. The recoveries were found 98% for PCNB and 97% for HCB compounds.

**Table 1:** Recoveries (%) of PCNB and HCB from spiked soil calculated by HPLC

**Spiked concentration of PCNB (gm kg**^-**1**^**)**	**Concentration (Mean ± SD) (gm kg**^-**1**^**)**	RSD (%)	Recovery (%)	**Spiked concentration of HCB (gm kg**^-**1**^**)**	**Concentration (Mean ± SD) (gm kg**^-**1**^**)**	RSD (%)	Recovery (%)
0.05	0.0489 ± 0.0016	3.21	97.80	0.05	0.0488 ± 0.0023	1.45	97.60
0.1	0.0979 ± 0.0025	2.84	97.79	0.1	0.0978 ± 0.0025	1.78	97.80
0.2	0.1968 ± 0.0016	4.12	98.40	0.2	0.1957 ± 0.0028	2.10	97.85
0.3	0.2976 ± 0.0018	1.83	99.20	0.3	0.2949 ± 0.0053	2.91	98.30
0.4	0.3938 ± 0.0013	2.13	98.45	0.4	0.3937 ± 0.0019	2.24	98.42

To determine any interference caused by the endogenous and soil component, a blank soil sample was analyzed. No interference was identified from the soil sample. The limit of detection (LOD) were also calculated by applying the 3s criterion equation: y-yb = 3sb, where as 'y' is lowest concentration signal and 'yb' is a blank signal, i.e. LOD corresponds to a signal equal to 3 times of standard deviation of the background noise. The detection limit for PCNB was found to be 0.0001 μg ml**^-^**^1 ^and 0.0003 μg ml**^-^**^1 ^for HCB. Similar exercise was carried out for both PCNB and HCB from MSM. Result indicated that extraction efficiency of PCNB and HCB from MSM was very close to the extraction efficiency as determined for the soil sample (data not shown).

### UV-vis absorption spectra and retention time of PCNB, HCB and their possible intermediates

Identification of any aromatic compound by HPLC can be performed by comparing the UV-vis absorption spectra and retention time with the authentic standards. The retention time and UV-vis absorption spectra of PCNB, HCB and their possible intermediates are given in the Table 2. The proposed method is also well suitable for the identification of chlorophenolics and chloroquinolics intermediates such as PCP, TeCP, TeCH, TCP, and DiCH.

**Table 2:** Details of retention times and corresponding UV-vis absorption spectra of PCNB, HCB and their possible intermediates determined by HPLC

Name of the compounds	Retention time (min)	UV-vis absorption spectra (nm)
PCNB	6.692	301.0
PeCA	3.532	221.1
TeCA	5.375	308.5
TCA	4.121	248.1
DCA	6.951	246.9
PCP	5.780	303.7
HCB	22.88	290.7
PeCB	16.31	287.1
TeCH	3.735	297.8
TeCB	9.746	229.3
TeCP	4.520	299.0
TCB	12.06	268.2
TeCNB	4.266	223.4
TCP	3.966	289.5
DCNB	3.728	222.2
DCB	4.976	284.8
DiCH	4.214	277.6
DCP	3.457	281.2

### Method validation

#### Determination of PCNB, HCB depletion rate and identification of metabolites

**Figure 1:**
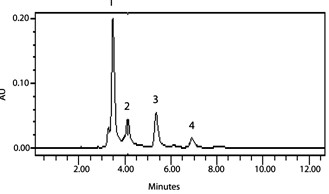
**Representative HPLC chromatogram of PCNB metabolites identified from anaerobic enriched soil sample**. Peak 1, pentachloroaniline (PeCA); Peak 2, tetrachloroaniline (TeCA); Peak 3, trichloroaniline (TCA); and Peak 4, dichloroaniline (DCA).

**Figure 2:**
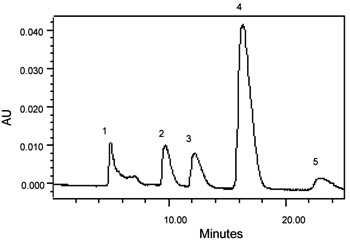
**Representative HPLC chromatogram of HCB and their putative intermediates identified from anaerobic enriched soil sample**. Peak 1, dichlorobenzene (DCB); Peak 2, trichlorobenzene (TCB); Peak 3, tetrachlorobenzene (TeCB); and Peak 4, pentachlorobenzene (PeCB); and Peak 5-hexachlorobenzene (HCB).

**Figure 3:**
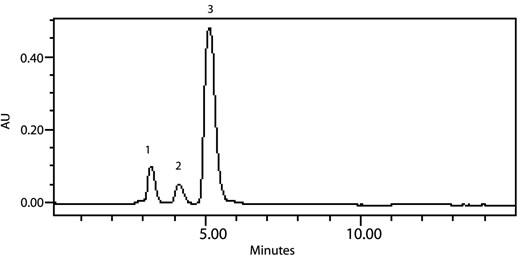
**Representative HPLC chromatogram of chlorophenolics and chlorohydroquinolics standards showing; Peak 1, dichlorohydroquinone (DiCH); Peak 2, tetrachlorohydroquinone (TeCH); and Peak 3-pentachlorophenol (PCP)**.

For validation of satisfactory results presented here, we applied this extraction and analytical method to determine PCNB, HCB depletion rates and also identification of metabolites from the PCNB and HCB enriched soil sample under anaerobic condition for up to a five months incubation period. Samples were drawn, extracted and analyzed at regular intervals by HPLC to determine the rate of depletion and appearance of intermediates. It was found that the rate of PCNB and HCB depletion increased with the time of incubation (data not shown). PCNB degradation intermediates viz; pentachloroaniline (PeCA), tetrachloroaniline (TeCA), trichloroaniline (TCA) and dichloroaniline (DCA) from anaerobic enriched soil sample have been identified by HPLC with the retention time 3.532, 5.375, 4.121, 6.951 min and UV-absorption spectra 246.9, 308.5, 248.1, 221.1 nm (Figure 1). There are few reports on degradation of PCNB by anaerobic mixed culture via reductive dechlorination under anaerobic condition [22,25]. Similarly, HCB degradation intermediates from anaerobic enrichment were identified as pentachlorobenzene (PeCB), tetrachlorobenzene (TeCB), trichlorobenzene (TCB) and dichlorobenzene (DCB) with the retention time 16.31, 9.746, 12.06, 4.976 min and UV-absorption spectra 287.1, 229.3, 268.2, 284.8 nm (Figure 2). The same HCB degradation metabolites have also been identified by reductive dechlorination in soil sediment [14]. There is one report on HCB degradation by *Nocardioides *sp. strain PD653, the identification of produced intermediates such as pentachlorophenol (PCP), tetrachlorohydroquinone (TeCH), 2, 6-dichlorohydroquinone (DiCH) performed by GC after acetylation with acetic anhydride [26]. In the proposed method of identification of such phenolic intermediates by HPLC, there is no need of derivatization. The retention time and UV-vis absorption spectra of chlorophenolic and chloroquinolic standards such as pentachlorophenol (PCP), tetrachlorohydroquinone (TeCH) and dichlorohydroquinone (DiCH) was found to 5.780, 4.214, and 3.735 minute and 303.7, 297.8, 277.6 nm (Figure 3).

## Discussion

Due to high electronegative properties of PCNB, HCB and their intermediates, the identification is possible by gas chromatography coupled with electron capture detector (GC-ECD) and mass-spectroscopy (GC-MS) [30,32,33]. Although, the detection efficiency by these methods is good, yet there are several drawbacks like the need to derivatize such as methylation, acetylation and pentafluorobenzylation and the loss of compounds by thermal decomposition [24,26,27,34]. Similarly, there are a few reports describing extraction procedures for PCNB and HCB but they are cumbersome as they required additional steps, of sample cleanup or sample enrichment followed by quantitative determination by liquid chromatographic analysis [24,35]. In the present work, solubility analysis was carried out which indicated that all compounds were highly soluble in ethyl acetate, except HCB which was soluble in hexane. Based on their solubility, ethyl acetate and hexane were used for extraction of PCNB and HCB respectively from the soil sample as well as from the MSM. The proposed liquid-liquid extraction and HPLC analysis procedure showed good recoveries of 98% with 0.0001 μg ml**^-^**^1 ^LOD for PCNB and 97% with 0.0003 μg ml**^-^**^1 ^LOD for HCB without the need to cleanup the extracted samples. In the proposed HPLC method there is no need for the derivatization such as methylation, acetylation or pentafluorobenzylation for the detection of chlorophenolic and chloroquinolic intermediates. The proposed liquid-liquid extraction and analytical method is one of the most suitable for the application to any toxic organochlorine residue determination from different environmental compartment such as soil, sediment and sludge.

## Conclusion

A rapid, sensitive and simple analytical method was developed for the determination of polychlorinated fungicides such as PCNB, HCB and their possible intermediates including chlorophenolic as well as chloroquinolic intermediates using HPLC techniques. The developed method gave good recoveries, good peaks resolution and sensitive detection levels without internal interference.

## Methods

### Chemicals, reagents and standard stocks

The standards of PCNB, HCB including their possible intermediates such as PeCA, PCP, TeCA, TCA, DCA, PeCB, TeCB, TeCH, TCB, TeCNB, TeCP), TCP, TCNB, DCP, DCNB, DCA, DiCH and DCB were purchased from Sigma Aldrich (St, Louis, MO, USA). Ethyl acetate, methanol, and hexane (HPLC grade) were obtained from Merck (Darmstadt, Germany). The stock solution (100 μg ml^-1^) of the above compounds was prepared in dimethyl sulphooxide (DMSO) of PCNB and in hexane of HCB. Stock solutions were diluted to obtain 10 μg ml^-1 ^working solutions. All chemicals, aqueous solutions and standards were filtered through 0.22 μm filter (Millipore, Milford, Mass., USA) and stored at 4°C. The composition of minimal salt medium (MSM) used for determining the extraction efficiency of PCNB and HCB compounds was the same as described by Fazlurrahman et al. [36].

### Sample preparation and fortification

Soil samples (collected locally) were air dried, homogenized, grounded, sieved and stored in stoppered glass flasks at 18°C. Fifteen gram soil sample was fortified by adding five working concentration of PCNB and HCB equivalent to 0.05, 0.1, 0.2, 0.3 and 0.4 mg kg**^-^**^1 ^respectively. The standard solution(s) was mixed in 20 ml MSM and added to the soil verifying that the solution covered the soil particles completely. After fortification, the samples were mixed with the help of glass rod followed by vortexing and kept in dark at room temperature for 24 h for equilibration. Since most of the microbial degradation of pesticide were also carried out in MSM, therefore the extraction efficiency of PCNB and HCB was also estimated from 10 ml MSM containing 0.5, 1.0, 2.0, 3.0 and 4.0 μg ml**^-^**^1 ^respective compounds.

### Extraction efficiency of PCNB and HCB compounds from different sources

For extraction, 15 g of the soil sample containing the standard compounds was blended with 45 ml of ethyl acetate for PCNB and 45 ml of hexane for HCB, vortexed vigorously for 10 min and centrifuged for 10 min at 15000 rpm. The upper organic phase was collected and evaporated to dryness in rotary evaporator at 45-50°C. Extraction was repeated three times and the residues were re-dissolved in 1 ml of their suitable solvent. The same procedure of extraction for the added PCNB and HCB to the MSM was performed by adding equal volume of ethyl acetate for PCNB and hexane for HCB. Similarly, the same extraction and analytical method were adapted for the extraction and analysis of PCNB, HCB degradation and metabolites identification from the anaerobic enriched soil sample. Finally, extraction efficiency of PCNB and HCB from spiked soil sample and MSM was calculated with the help of following equation:

Percent extraction efficiency=(Peak area of extracted sample for XmM∕Peak area of standard sample for XmM)×100

### Chromatographic apparatus and conditions

The liquid chromatographic system consisted of Water-HPLC 600 and multi-solvent delivery system pump with Waters 996 photodiode array detector (Waters, Corporation, Milford, MA, USA). A reverse phase Waters Spherisorb 5 μm C_18 _(150 × 4.6 mm) column (Waters, Corporation, Milford, MA, USA) was used as the stationary phase. The separation of analytes on HPLC was conducted by using RPC_18 _column with mobile phase methanol: water (96:4, v/v) at a column temperature of 25°C and a flow rate of 1.0 ml min**^-^**^1^. The injection volume was 5 μl. The detection of the analytes was realized by measuring the UV-absorption with PDA detector at 300 nm.

## Abbreviations

(CACs): Chlorinated aromatic compounds; (PCNB): Pentachloronitrobenzene; (HCB): Hexachlorobenzene; (PeCA): pentachloroaniline; (TeCA): tetrachloroaniline; (TCA): trichloroaniline; (DCA): dichloroaniline; (CA): chloroaniline; (PCP): pentachlorophenol; (PeCB): pentachlorobenzene; (TeCB): tetrachlorobenzene; (TCB): trichlorobenzene; (DCB): dichlorobenzene; (TeCH): tetrachlorohydroquinone; and (DiCH): dichlorohydroquinone; (TeCP): tetrachlorophenol; (TCP): trichlorophenol; (DCP): dichlorophenol; (HPLC): high performance liquid chromatography; (PeCA): pentachloroaniline; (TeCA): tetrachloroaniline; (TCA): trichloroaniline; and (DCA): dichloroaniline; (TeCB): tetrachlorobenzene; and (GC-ECD): gas chromatography coupled with electron capture detector; and (GC-MS): mass-spectroscopy; (MSM): minimal salt medium.

## Authors' contributions

FK and DP design the study, carried out the experiments, analyzed the experimental data and drafted the manuscript. RKJ conceived the project, coordinated it and refined the manuscript. All authors have read and approved the final manuscript.
